# Clinically relevant factors associated with quantitative optical coherence tomography angiography metrics in deep capillary plexus in patients with diabetes

**DOI:** 10.1186/s40662-019-0173-y

**Published:** 2020-02-03

**Authors:** Fang Yao Tang, Erica O. Chan, Zihan Sun, Raymond Wong, Jerry Lok, Simon Szeto, Jason C. Chan, Alexander Lam, Clement C. Tham, Danny S. Ng, Carol Y. Cheung

**Affiliations:** 1 Department of Ophthalmology and Visual Sciences, The Chinese University of Hong Kong, Hong Kong Special Administrative Region, Kowloon, China; 2 Hong Kong Eye Hospital, Hong Kong Special Administrative Region, Kowloon, China; 3 CUHK Eye Centre, Hong Kong Eye Hospital, 147K Argyle Street, Kln, Kowloon, Hong Kong China

**Keywords:** Optical coherence tomography angiography, Diabetic retinopathy, Deep capillary plexus, Visual acuity

## Abstract

**Background:**

To test clinically relevant factors associated with quantitative artifact-free deep capillary plexus (DCP) metrics in patients with diabetes mellitus (DM).

**Methods:**

563 eligible eyes (221 with no diabetic retinopathy [DR], 135 with mild DR, 130 with moderate DR, and 77 with severe DR) from 334 subjects underwent optical coherence tomography-angiography (OCT-A) with a swept-source OCT (Triton DRI-OCT, Topcon, Inc., Tokyo, Japan). Strict criteria were applied to exclude from analysis those DCP images with artifacts and of poor quality, including projection artifacts, motion artifacts, blurriness, signal loss, B-scan segmentation error, or low-quality score. A customized MATLAB program was then used to quantify DCP morphology from the artifact-free DCP images by calculating three metrics: foveal avascular zone (FAZ), vessel density (VD), and fractal dimension (FD).

**Results:**

166 (29.5%) eyes were excluded after quality control, leaving in the analysis 397 eyes (170 with no DR, 101 with mild DR, 90 with moderate DR, 36 with severe DR) from 250 subjects. In the multiple regression models, larger FAZ area was associated with more severe DR (β = 0.687; *p* = 0.037), shorter axial length (AL) (β = − 0.171; *p* = 0.003), thinner subfoveal choroid thickness (β = − 0.122; *p* = 0.031), and lower body mass index (BMI) (β = − 0.090; *p* = 0.047). Lower VD was associated with more severe DR (β = − 0.842; *p* = 0.001), shorter AL (β = 0.107; *p* = 0.039), and poorer visual acuity (VA) (β = − 0.133; *p* = 0.021). Lower FD was associated with more severe DR (β = − 0.891; *p* < 0.001) and with older age (β = − 0.142; *p* = 0.004).

**Conclusions:**

Quantitative artifact-free DCP metrics are associated with VA, DR severity, AL, subfoveal choroidal thickness, age, and BMI in diabetic patients. The effects of ocular and systemic factors should be considered for meaningful interpretations of DCP changes in DM patients.

## Background

Diabetic retinopathy (DR) is a common microvascular complication of diabetes mellitus (DM). Diabetic macular ischemia (DMI), a clinical feature of DR characterized by retinal capillary loss and enlargement of the foveal avascular zone (FAZ), is a major cause of vision loss in DM patients [1]. With the advances in optical coherence tomography angiography (OCT-A), depth-resolved visualization of individual vascular layers (e.g., superficial capillary plexus [SCP] and deep capillary plexus [DCP]) and studying DMI without intravenous dye injection are now possible [2, 3].

Recent OCT-A studies showed that in DM patients, the DCP suffers more severe microvascular damage than the SCP, indicating that DCP has a more pronounced vessel loss and a stronger correlation with functional deficit from DMI [4, 5]. Despite this, OCT-A artifacts are common, particularly the projection artifacts, which are the fluctuating shadows cast by the flowing blood cells in the overlying retinal vessels projecting to the deeper layers [6, 7]. However, the preexisting studies, which apply OCT-A to investigate the correlation of quantitative DCP metrics with DR and visual acuity (VA), have not effectively addressed in their findings the issue of projection artifacts [8–11]. Failure to consider this disruption in the vessel networks affects the accurate interpretation of DCP. Furthermore, there is a lack of studies examining whether diabetes-associated, systemic (e.g., hemoglobin A1c level), and ocular factors can influence DCP metrics. Understanding the associated factors is important, as this will help improve the interpretation of DCP when examining the correlation between DCP metrics and DR and DMI in DM patients [12], particularly in employing DCP metrics as diagnostic or prognostic markers in future clinical practice.

In this study, we aimed to investigate the influence of diabetes-associated, systemic, and ocular factors on quantitative DCP metrics (FAZ area, vessel density [VD], and fractal dimension [FD]) in a cohort of DM patients. Before our investigation, we applied stringent quality control criteria to select the appropriate DCP images for analysis in order to minimize effect from image artifacts including projection artifacts.

## Materials and methods

### Subjects

We conducted a cross-sectional observational study of DM patients recruited from January 2016 through July 2017 at CUHK Eye Centre, Hong Kong Eye Hospital. Inclusion criteria for study eyes included [1] patients with type 1 or type 2 DM [2]; spherical refractive error within the range of − 8.5 to + 4.0 diopter (D) with less than 5.0 D of cylinder; and [3] VA not worse than Snellen 20/200. Exclusion criteria for study eyes included [1] prior retinal surgery, intraocular surgery, intravitreal injection, and retinal laser photocoagulation [2]; eye conditions which interfere with imaging and VA (e.g., dense cataract, corneal ulcer) [3]; glaucoma [4]; eye pathology unrelated to DM (e.g., wet age-related macular degeneration, epiretinal membrane and other maculopathy); and [5] patients who failed to cooperate when taking OCT-A images (e.g., fail to fixate their eyes for 7–8 s).

This study was conducted in accordance with the 1964 Declaration of Helsinki and was approved by the Kowloon Central/East Research Ethics Committee. Written informed consent were obtained from all subjects.

### OCT-A imaging

All recruited subjects underwent OCT-A with a swept-source OCT (Triton DRI-OCT, Topcon, Inc., Tokyo, Japan). Volumetric OCT scans centered on the fovea were obtained with a scan area of 3 mm × 3 mm containing 320 × 320 A-scans. The built-in software (IMAGEnet6, v1.23.15008, Basic License 10) was used to identify SCP and DCP. The DCP delineated by this software was 15.6 μm below the junction between inner plexiform and inner nuclear layer (IPL/INL) to 70.2 μm below IPL/INL.

### OCT-A image quality control

Before quantitative analysis, a single reader (EOC) carefully evaluated each DCP image and OCT cross-sectional B-scan at the CUHK Ocular Reading Centre. The reader was masked to all patients’ characteristics.

#### Assessment of projection artifacts

Stringent criteria were applied to exclude those DCP images with projection artifacts i.e., a result of overriding blood vessel shadow from SCP appearing erroneously at DCP. A two-step method was applied to identify projection artifacts on DCP images. First, SCP and DCP images taken at the same scan by OCT-A were compared side by side. Locations at which major and large vessels appear on the SCP image were traced along the same locations on the DCP image. This was to identify any continuous vessels with morphology and caliber similar to SCP appearing on the DCP image at the same site since these were the potential projection artifacts. Second, the potential projection artifacts identified would be studied for their morphology to decide whether it is likely to reflect the blood vessels from the overriding SCP. It has been established that both SCP and DCP have a distinctive morphology [13]. Vessels at DCP have a vortex-like capillary arrangement [14] with capillaries radially converged toward an epicenter known as “vortex,” and are composed of polygonal units. The deep capillary vortexes are found along the venules at SCP and drain into the superficial venules [14, 15]. Additional file 2**:** Figure S1 shows examples that compare the different morphologies of SCP and of DCP. If the potential projection artifact identified at DCP was a continuous vessel which did not appear as a series of vortexes with converged capillaries and polygonal units, it was to be taken as a projection artifact on DCP. The DCP images were excluded when projection artifacts were identified.

#### Assessment of other OCT-A artifacts

**Fig. 1:**
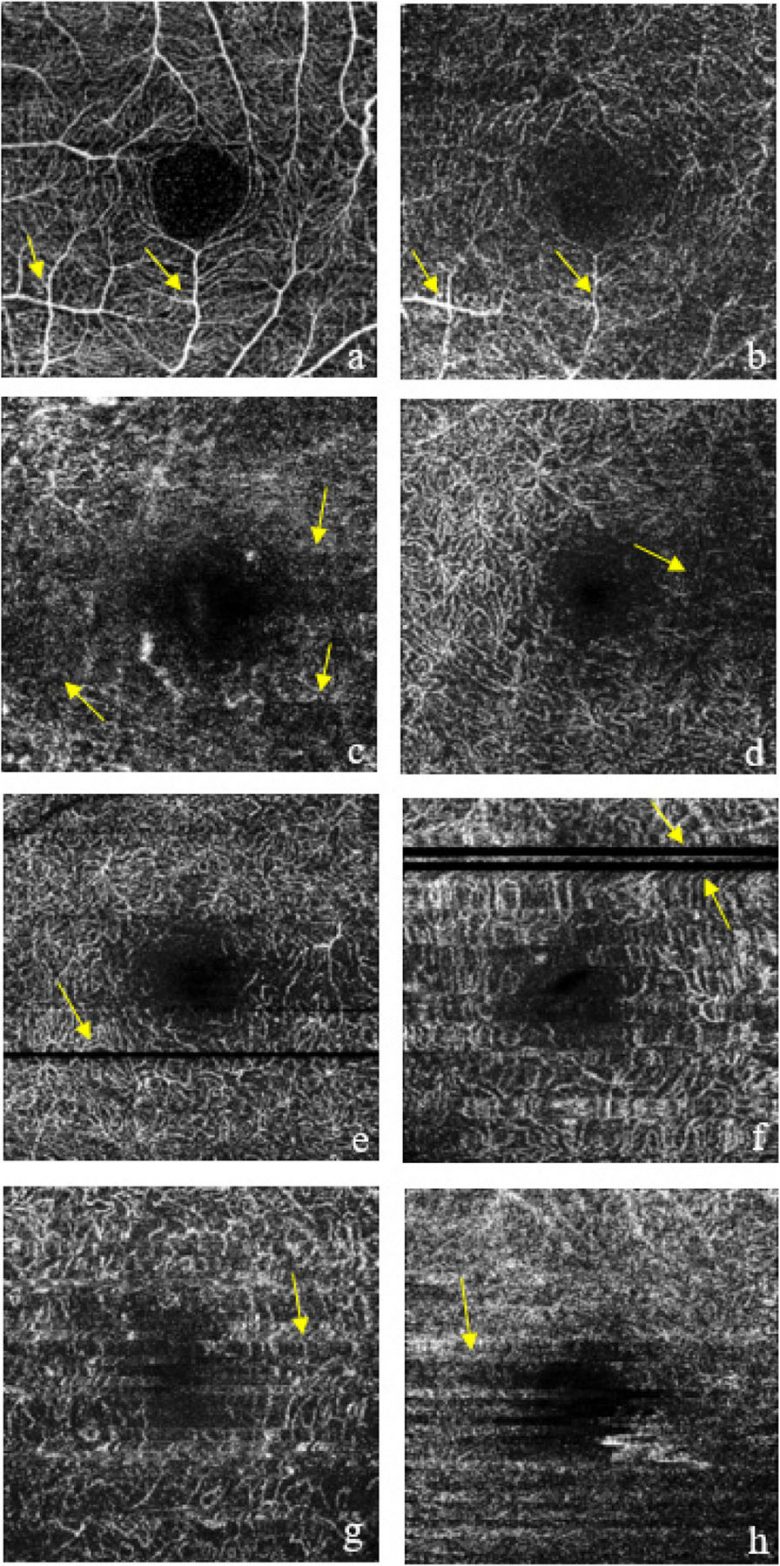
Examples of excluded DCP images during quality control process: projection artifacts (**a**, **b**); blurred images (**c**, **d**); signal loss due to blinking (**e**, **f**); and motion artifacts (**g**, **h**)

Strict criteria were also applied to exclude images from the analysis for them having a quality score below 40, motion artifacts (e.g., vessel discontinuity or significant residual motion lines), blurry images (e.g., due to media opacity or axial movement), signal loss (e.g., due to blinking), or the fovea poorly concentrated and deviated from the center. Images with segmentation error were also excluded; they are defined as any detectable deviation from the expected boundary for any B-scan [16]. Examples of excluded images are shown in Fig. 1.

### Quantification of deep capillary network

The DCP images were imported into an automated customized MATLAB program used for SCP previously for image processing and analysis [17]. The parameters for denoising were tested and fine-tuned for analyzing the DCP images. Three DCP metrics were calculated: FAZ area, VD, and FD. FAZ area (mm^2^) was calculated by counting in scale the total number of pixels within the region. VD was calculated as the percentage of area not defined as non-perfusion regions (dark areas from the binarized image larger than 0.02 mm^2^) over the total area within the parafoveal region (an annulus with an outer diameter of 3 mm and an inner diameter of 1 mm). The binarized image was also skeletonized and FD was calculated by the box-counting method.

### Reliability assessment

Intra-session (repeated OCT-A imaging in the same visit) and inter-session (repeated OCT-A imaging in another visit within 2 weeks) reliability assessments of FAZ area, VD and FD, measured using our customized program, were conducted in one randomly selected eye from a subset of 29 randomly selected DM subjects. There was a 5-min interval between the two scans during a single visit, and the subject was invited for another visit for OCT-A scan within 2 weeks for assessing inter-session reproducibility.

### Measurement of diabetes-associated, ocular and systemic factors

The diabetes-associated factors included the duration of diabetes obtained from interview-based questionnaires and the level of serum glycosylated hemoglobin (HbA1c) by reviewing the recent fasting blood test results in the patient’s medical record.

The ocular factors included DR severity, presence of diabetic macular edema (DME), VA, axial length (AL), central subfield thickness (CST), average ganglion cell-inner plexiform layer (GC-IPL) thickness, and subfoveal choroidal thickness. Dilated biomicroscopic fundus examination was performed by retinal specialists to assess DR severity and the presence of DME, according to the International Clinical Diabetic Retinopathy and Diabetic Macular Edema Disease Severity Scales. DR severity was categorized into no DR, mild, moderate and severe non-proliferative DR (NPDR), or proliferative diabetic retinopathy (PDR). VA was obtained in both eyes for all subjects (with subjective refraction, or corrected by glasses or contact lens, or through a pinhole) using a Snellen chart at a distance of 6 m, with the non-tested eye covered. The best score for VA was recorded for each eye using metric notation from the Snellen chart, and converted to logarithm of the minimum angle of resolution (logMAR). AL was measured with a non-contact partial coherence laser interferometry (IOL Master, Carl Zeiss Meditec, Dublin, US). Five measurements were taken, and the mean was used in the analysis. CST and average GC-IPL thickness were measured with Cirrus HD-OCT (Carl Zeiss Meditec Inc., Dublin, CA, USA). Subfoveal choroidal thickness was obtained from horizontal scan with the Triton SS-OCT device, measured by the built-in caliber. The systemic factors included age, body mass index (BMI), systolic blood pressure (SBP), diastolic blood pressure (DBP), total cholesterol level, and low-density lipoprotein (LDL) cholesterol level. SBP and DBP were measured with a digital automatic blood pressure monitor (model Avant 2120; Nonin Medical, Inc., Plymouth, MN, USA). Levels of lipids and the most recent fasting blood test results were reviewed from patients’ medical records.

### Statistical analysis

All statistical analyses were performed using IBM SPSS statistics version 23.0. Generalized estimating equations (GEE) models were used to examine the associations of the diabetes-associated, ocular and systemic factors (independent variables) with DCP metrics (dependent variables), namely FAZ area, VD and FD. Continuous independent variables were first standardized (i.e., per standard deviation [SD] increase). Then, we performed a univariate linear regression analysis to determine the associations between diabetes-associated, ocular, and systemic factors with the DCP metrics. Factors showing significant association in the univariate analysis were included in the multiple regression analyses.

In the second part of the analysis, we excluded eyes with DME and repeated the above analysis, as fluid from DME may be trapped between retinal layers, and thus obscuring the vasculature reflected from the DCP and possibly being mistaken as non-perfusion on DCP images [6]. Analysis with eyes without DME was therefore performed to remove the potential artifact from overlying retinal cystic changes by DME, which may overestimate the extent of non-perfusion at DCP, and thereby affecting the associations identified between its metrics and the determinants.

## Results

563 eyes from 334 subjects were eligible for this study. Quality control was implemented to exclude DCP images with artifacts. 397 eyes from 250 subjects were included in the analysis after the quality check. Eyes were excluded mainly for the following reasons: projection artifacts (*n* = 53), blurriness of images (*n* = 67), motion artifacts (*n* = 27), signal loss (*n* = 10), low quality score (*n* = 8), and poor concentration (*n* = 1).

Table 1 shows the demographics and the clinical characteristics of the included and excluded eyes/subjects. Among the included eyes, there were 170 eyes (35.3%) without DR, 101 eyes (25.4%) with mild NPDR, 90 eyes (22.7%) with moderate NPDR, and 36 eyes (9.07%) with severe NPDR or PDR. 57 eyes (14.4%) also had DME. Among the included subjects, 46% were females. The mean age was 60.69 years (SD = 13.2), with a mean DM duration of 12.9 years (SD = 9.07) and mean HbA1c level of 7.48% (SD = 1.33). Compared with included eyes/subjects, the excluded eyes/subjects were more likely to have DME, poorer VA, thinner GC-IPL, thinner subfoveal choroidal thickness, older age, higher blood pressure, and lower total cholesterol level. We also compared the demographics and the clinical characteristics of the eyes/subjects excluded due to projection artifacts with those eyes/subjects without any OCT-A image artifacts as shown in the Additional file 1: Table S1. The eyes/subjects with projection artifacts were more likely to have more severe DR, poorer VA, older age, higher blood pressure, and to manifest a history of coronary artery disease.

**Table 1:** Clinical characteristics of included and excluded participants

	Included	Excluded	*P*-value
	By eyes (*n* = 397)	By eyes (*n* = 166)	
Diabetic retinopathy severity (no/ mild/ moderate/ severe)	170/ 101/ 90/ 36 (43%/ 25%/ 23%/ 9%)	51/ 34/ 40/ 41 (31%/ 20%/24%/25%)	0.053
Presence of diabetic macular edema	57 (14.4%)	60 (36.1%)	0.005
LogMAR, per unit	0.14 (0.15)	0.26 (0.24)	< 0.001
Axial length (mm)	23.88 (1.34)	23.88 (1.33)	0.993
Central subfield thickness (μm)	258.2 (47.4)	267.0 (73.2)	0.194
Subfoveal choroidal thickness (μm)	208.58 (78.13)	188.82 (75.48)	0.011
Average GC-IPL thickness (μm)	80.2 (10.9)	77.2 (12.0)	0.012
Average peripapillary RNFL thickness (μm)	97.97 (65.89)	91.24 (14.48)	0.060
	By subjects (*n* = 250)	By subjects (*n* = 83)	
Gender, Female	115 (46%)	37 (44.6%)	0.317
Age (years)	60.7 (13.2)	65.7 (11.1)	0.002
Duration of diabetes (year)	12.9 (9.07)	11.3 (8.76)	0.182
Body mass index (kg/m^2^)	26.2 (4.19)	25.2 (4.70)	0.119
Systolic blood pressure (mmHg)	137.8 (20.4)	147.0 (20.9)	0.001
Diastolic blood pressure (mmHg)	78.1 (10.5)	77.3 (11.7)	0.626
Pulse Pressure (mmHg)	60.7 (18.40)	69.7 (17.6)	< 0.001
HbA1c (%)	7.48 (1.33)	7.50 (1.67)	0.958
Fasting glucose (mmol/L)	7.87 (3.33)	7.95 (2.38)	0.860
Total cholesterol (mmol/L)	4.34 (0.95)	3.99 (0.75)	0.003
LDL cholesterol (mmol/L)	2.31 (0.77)	2.14 (0.56)	0.068
HDL cholesterol (mmol/L)	1.37 (0.50)	1.30 (0.39)	0.287
Creatinine (μmol/L)	91.8 (71.13)	92.7 (61.9)	0.918
History of stroke	10 (4%)	3 (3.61%)	0.998
History of coronary artery disease	35 (14%)	16 (19.3%)	0.140

*GC-IPL=* ganglion cell inner plexiform layer; *LogMAR=* logarithm of the minimum angle of resolution; *RNFL=* retinal nerve fiber layer; *HbA1c=* hemoglobin A1c; *LDL=* low-density lipoprotein; *HDL=* high-density lipoproteins

In the reliability analysis, the intra-class correlation coefficients (ICCs) for intra-session repeatability of FAZ area, VD and FD were 0.672 (95% CI: 0.404–0.833), 0.505 (95% CI: 0.169–0.736) and 0.945 (95% CI: 0.884–0.974), respectively; while the ICCs for inter-session reproducibility of FAZ area, VD and FD were 0.633 (95% CI: 0.346–0.811), 0.494 (95% CI: 0.155–0.729) and 0.957 (95% CI: 0.910–0.980), respectively.

Multiple regression models of FAZ area, VD, FD at DCP with the variables indicating significant associations in the univariate analysis are shown in Table 2. Larger FAZ area was associated with more severe DR (β = 0.687, 95% CI: 0.041–1.333, *p* = 0.037); shorter AL (β = − 0.171, 95% CI: − 0.282 to 0.059, *p* = 0.003); thinner subfoveal choroidal thickness (β = − 0.122, 95% CI: − 0.232 to 0.011, *p* = 0.031); and lower BMI (β = − 0.090, 95% CI: − 0.180 to 0.001, *p* = 0.047). Lower VD was associated with more severe DR (β = − 0.842, 95% CI: − 1.322 to 0.363, *p* = 0.001); shorter AL (β = 0.107, 95% CI: 0.005–0.209, *p* = 0.039); and poorer VA (β = − 0.133, 95% CI: − 0.245 to 0.020, *p* = 0.021). Lower FD was associated with more severe DR (β = − 0.891, 95% CI: − 1.331 to 0.451, *p* < 0.001); thinner average GC-IPL (β = 0.113, 95% CI: 0.007–0.220, *p* = 0.037); lower BMI (β = 0.035, 95% CI: 0.005–0.149, *p* = 0.035); and older age (β = − 0.142, 95% CI: − 0.239 to 0.044, *p* = 0.004).

**Table 2:** Multiple regression models of (a) foveal avascular zone area, (b) vessel density, (c) fractal dimension with variables that showed significant associations in univariate analysis

	Beta coefficient	Standard error	95% CI	P-value
(a) Foveal Avascular Zone (FAZ) Area				
Severity of DR				
Severe NPDR or PDR vs. No DR	0.687	0.330	0.041 to 1.333	0.037
Moderate NPDR vs. No DR	0.103	0.148	−0.187 to 0.392	0.487
Mild NPDR vs. No DR	0.221	0.130	−0.034 to 0.475	0.089
Presence of DME	0.138	0.171	− 0.196 to 0.473	0.418
LogMAR, per SD increase	0.102	0.058	−0.012 to 0.215	0.079
Axial length, per SD increase	−0.171	0.057	−0.282 to − 0.059	0.003
Subfoveal choroidal thickness, per SD increase	−0.122	0.056	−0.232 to − 0.011	0.031
Body mass index, per SD increase	−0.090	0.046	−0.180 to − 0.001	0.047
Systolic blood pressure, per SD increase	0.077	0.067	−0.053 to 0.207	0.246
Age, per SD increase	0.051	0.053	−0.052 to 0.154	0.336
(b) Vessel Density (VD)				
Severity of DR				
Severe NPDR or PDR vs. No DR	−0.842	0.245	−1.322 to − 0.363	0.001
Moderate NPDR vs. No DR	−0.249	0.145	−0.532 to 0.035	0.085
Mild NPDR vs. No DR	−0.345	0.124	−0.588 to − 0.101	0.006
Presence of DME	−0.160	0.167	−0.488 to 0.168	0.340
Axial length, per SD increase	0.107	0.052	0.005 to 0.209	0.039
LogMAR, per SD increase	−0.133	0.057	−0.245 to − 0.020	0.021
Subfoveal choroidal thickness, per SD increase	0.104	0.056	−0.005 to 0.213	0.062
Body mass index, per SD increase	0.073	0.054	−0.033 to 0.178	0.176
Systolic blood pressure, per SD increase	−0.030	0.057	−0.143 to 0.082	0.595
Age, per SD increase	−0.078	0.058	−0.192 to 0.036	0.182
(c) Fractal Dimension (FD)				
Severity of DR				
Severe NPDR or PDR vs. No DR	−0.891	0.225	−1.331 to − 0.451	< 0.001
Moderate NPDR vs. No DR	−0.357	0.128	−0.607 to − 0.107	0.005
Mild NPDR vs. No DR	−0.307	0.109	−0.521 to − 0.093	0.005
LogMAR, per SD increase	−0.074	0.055	−0.183 to 0.034	0.180
Average GC-IPL, per SD increase	0.113	0.054	0.007 to 0.220	0.037
Body mass index, per SD increase	0.077	0.037	0.005 to 0.149	0.035
Systolic blood pressure, per SD increase	0.062	0.045	−0.026 to 0.151	0.168
Age, per SD increase	−0.142	0.050	−0.239 to − 0.044	0.004

*CI=* confidence interval; *SD=* standard deviation; *DR=* diabetic retinopathy; *NPDR=* non-proliferative diabetic retinopathy; *PDR=* proliferative diabetic retinopathy; *DME=* diabetic macular edema; *GC-IPL=* ganglion cell inner plexiform layer; *LogMAR=* logarithm of the minimum angle of resolution

After excluding eyes with DME, the associations between DCP metrics were largely similar, except that the association between FD and average GC -IPL no longer existed (Table 3).

**Table 3:** Multiple regression models of (a) foveal avascular zone area, (b) vessel density, (c) fractal dimension with variables that showed significant associations in univariate analysis, excluding eyes without DME

	Beta coefficient	Standard error	95% CI	P-value
(a) Foveal Avascular Zone (FAZ) Area				
Severity of DR				
Severe NPDR or PDR vs. No DR	0.381	0.379	− 0.363 to 1.124	0.316
Moderate NPDR vs. No DR	0.157	0.157	− 0.152 to 0.465	0.319
Mild NPDR vs. No DR	0.256	0.143	−0.024 to 0.535	0.073
LogMAR, per SD increase	0.109	0.063	−0.014 to 0.233	0.083
Axial length, per SD increase	−0.201	0.057	−0.313 to − 0.089	< 0.001
Subfoveal choroidal thickness, per SD increase	−0.131	0.062	−0.252 to − 0.010	0.035
Body mass index, per SD increase	−0.083	0.049	−0.139 to 0.014	0.094
Systolic blood pressure, per SD increase	0.065	0.068	−0.067 to 0.198	0.335
Age, per SD increase	0.064	0.056	−0.046 to 0.174	0.253
>(b) Vessel Density (VD)				
Severity of DR				
Severe NPDR or PDR vs. No DR	−0.618	0.275	−1.158 to − 0.079	0.025
Moderate NPDR vs. No DR	−0.313	0.154	−0.614 to − 0.012	0.042
Mild NPDR vs. No DR	−0.325	0.133	−0.586 to − 0.064	0.015
Axial length, per SD increase	0.0130	0.056	0.021 to 0.239	0.019
LogMAR, per SD increase	−0.125	0.056	−0.235 to − 0.014	0.027
Subfoveal choroidal thickness, per SD increase	0.125	0.059	0.010 to 0.240	0.034
Body mass index, per SD increase	0.081	0.060	−0.037 to 0.199	0.177
Systolic blood pressure, per SD increase	−0.040	0.061	−0.159 to 0.078	0.504
Age, per SD increase	−0.095	0.062	−0.216 to 0.026	0.123
(c) Fractal Dimension (FD)				
Severity of DR				
Severe NPDR or PDR vs. No DR	−0.810	0.201	−1.204 to − 0.417	< 0.001
Moderate NPDR vs. No DR	−0.339	0.191	−0.714 to 0.036	0.076
Mild NPDR vs. No DR	−0.310	0.126	−0.556 to − 0.064	0.014
LogMAR, per SD increase	−0.023	0.095	−0.208 to 0.162	0.808
Average GC-IPL, per SD increase	−0.006	0.045	−0.095 to 0.082	0.893
Body mass index, per SD increase	0.099	0.045	0.011 to 0.187	0.028
Systolic blood pressure, per SD increase	0.033	0.054	−0.073 to 0.140	0.540
Age, per SD increase	−0.166	0.055	−0.275 to − 0.058	0.003

*DME=* diabetic macular edema; *CI=* confidence interval; *SD=* standard deviation; *DR=* diabetic retinopathy; *NPDR=* non-proliferative diabetic retinopathy; *PDR=* proliferative diabetic retinopathy; *GC-IPL=* ganglion cell inner plexiform layer; *LogMAR=* logarithm of the minimum angle of resolution

**Fig. 2:**
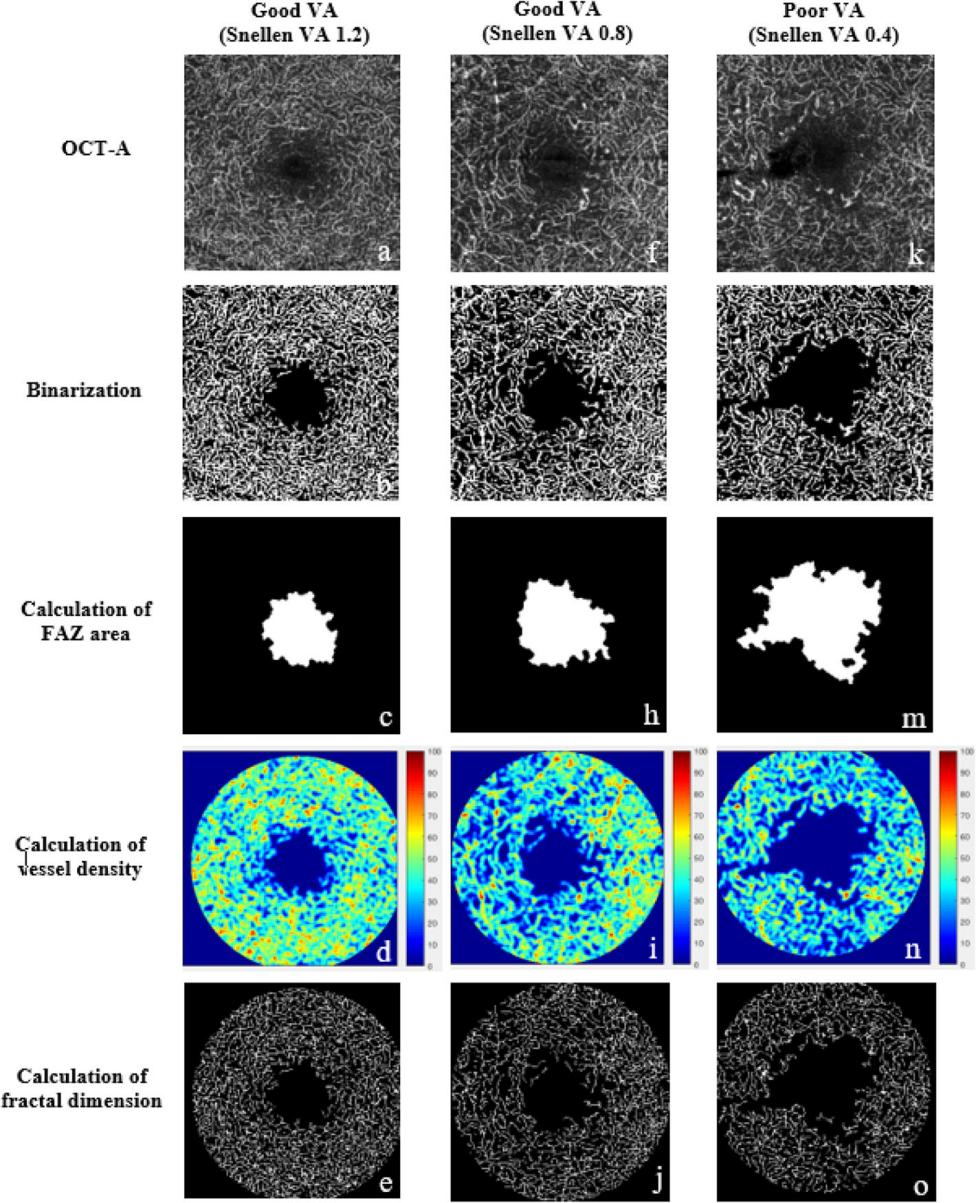
Examples of the quantification of deep capillary plexus using our customized program in patients with good (**a-j**) and poor (**k-o**) Snellen visual acuity. OCT-A metrics including foveal avascular zone (FAZ) area, vessel density (VD), and fractal dimension (FD) are calculated automatically

Figure 2 showed examples of DCP quantification using our customized program in patients with good and poor VD.

## Discussion

An advantage of OCT-A is the visualization of deeper retinal vascular plexuses via a layer-by-layer analysis, previously impossible with fluorescein angiography. However, artifacts, particularly projection artifacts, limits the accurate interpretation of DCP vasculature. Despite efforts in resolving the projection artifacts, the projection-resolved OCT-A algorithm still cannot remove the projection artifacts entirely, particularly those from the larger vessels [18–20]. For these limitations, our study resorted to enforcing stringent criteria to minimize image artifacts. We found that the DCP metrics were independently and significantly associated with VA, DR severity, AL, subfoveal choroidal thickness, age, and BMI in DM patients.

Decreased VD in DCP was found to be associated with reduced VA, suggesting that VD in DCP may reflect the degree of capillary loss in DM vision loss. There exist, albeit limited, consistent data to support the correlation between reduced VD and poorer VA in DCP [21, 22]. Samara et al. found a positive correlation between VA and FAZ area in both SCP and DCP for both healthy and DR eyes. Dupas et al. suggested that VA in DM patients depended mainly on VD of DCP and that VD reduction in DCP alone sufficiently results in visual loss but not if VD reduces only in SCP [21]. This further proves that there is an important association between VA and DCP – absent in VD of SCP [23]. DCP is responsible for 10–15% of the oxygen supply to photoreceptors and for the metabolic needs of photoreceptor synapses and axon terminals in the outer plexiform layer [24]. Considering that the compromise of photoreceptors would ultimately compromise VA, the DCP being first affected in DM with subsequent loss of photoreceptor function, implies that VA is associated with VD loss in the DCP, and that the ability to estimate and predict VA through OCT-A measurement will facilitate DR management and monitor the patient’s course of the disease.

DR severity was furthermore associated with larger FAZ area, lower VD, and lower FD – both before and after adjustment, with the largest effect seen in FD. Presence of DME was associated with all DCP metrics before adjusting for potential confounders but not afterwards. Our findings confirmed previous OCT-A studies on DCP assessment [22, 25–27], further supporting our conclusion that the degree of retinal microvascular damage resulting from hyperglycemia can be quantified and inferred by an enlarged avascular area, reduced VD, and a less complicated vessel network measured by OCT-A. The changes become more significant with more severe DR. While DR severity was associated with all DCP metrics, only the most severe DR was associated with increased FAZ area in multivariable analysis. It may be due to the high variability of FAZ size itself even among normal individuals, so the FAZ size of early DR may overlap with the normal eyes depending on their baseline FAZ size. Findings showing consistent correlations between DR severity and DCP/SCP metrics [28, 29] make the non-invasive OCT-A a potentially useful tool for identifying early microvascular changes in diabetic eyes, with the added advantage of detailed information regarding the individual layers of retinal capillaries.

Similar to a previous report [30] we found a longer AL to be associated with a smaller FAZ area in DCP [17, 30, 31], and increased VD. However, opposite results were reported by another group [32], potentially due to the stretching of the macular retina with eyeball elongation. Our results may be explained by ocular magnification as well, where longer AL increased the distance between the measured DCP, and thus the area between vessels appeared smaller, resulting in larger VD, similar to FAZ area [31, 32]. Although an image size correction method for AL was proposed [31], it was validated in the SCP only. Therefore, the method was not applied in this study. In addition, a thinner subfoveal choroid was observed in our cohort to be associated with enlarged FAZ and decreased VD. Previous swept-source OCT studies have observed an association between reduced choroidal thickness and volume with more advanced stages of DR [33, 34], suggesting that choroidal vessel abnormalities may occur simultaneously with or as a result of DR [35]. As enlarged FAZ and decreased VD are also associated with DR severity, our findings were in line with these previous studies.

Except for age and BMI, other systemic variables did not influence DCP metrics. In addition, there were no significant correlations between DCP metrics with HbA1c level and the duration of diabetes, the two diabetes-associated factors. Aging is known to be associated with the loss of complexity in organ structures of the human body. In our DM cohort, we found that an older age was associated with reduced FD. This was consistent with recent findings in SCP [30] and previous results relating to aging impacts on FD measured from retinal photographs in the general population [36]. We also observed that increased BMI was associated with increased FD and decreased FAZ area in DCP – explicable by the changes in vascular structure in obesity, which includes thickened basement membranes, increased vascular diameter, and stiffened resistance arterioles while the lumen size reduces. The increased diameter and thickening in the case of increased BMI may result in an increased occupancy of vessels in the OCT-A images, resulting ultimately in increased FD and decreased FAZ area [37]. However, caution is required for this interpretation because the underlying mechanism remains unclear.

In this study, DCP image was delineated 15.6 μm below the junction between IPL/INL to 70.2 μm below IPL/INL, using the built-in software (IMAGEnet6). However, the definitions of DCP vary in different algorithms. For example, the spectral-domain 70 kHz OCT instrument (AngioVue, RTVue-XR; Optovue) takes DCP as 15 to 70 μm below the IPL. The difference in segmentation of capillary plexuses at the retina may include intermediate capillary plexus (ICP) into the measurement of DCP in some OCT instruments. There are multiple vasculature network layers at retina, and the ICP is denser than other capillary plexuses while the DCP is largely flat and planar with closed vascular loops [38]. The different morphologies between ICP and DCP affect the metrics measured and the potentially different correlations with the ocular and systemic factors in diabetic patients. Furthermore, we did not observe any correlations between OCT-A metrics in DCP and GC-IPL. To date, the exact relationship between diabetic retinal microvascular alteration and neurodegeneration is not fully understood. Kim et al. found significant associations between GC-IPL thickness and FAZ area or VD in patients with DM but without DR [39], suggesting that neuroretinal degeneration occurs at an early stage of DM [39–41]. However, Carnevali et al. reported that there were no significant differences of GCL thickness, but only a significant reduction of VD in DCP in patients with type 1 diabetes when compared with the control group [5].

Our study has several strengths, including the adoption of strict criteria to minimize image artifacts, the adoption of standardized image acquisition protocol, the prospective study design, and the consideration of a wide range of diabetes-associated, ocular and systemic factors. However, there are several limitations. First, 29.5% of eligible images were excluded from the final analysis because of its artifacts, leading possibly to selection bias in subject sampling. Second, the intra-session and inter-session reliability of measurement of DCP metrics were generally lower compared with that of SCP, except for the FD measurement. The ICC of intra-session measurement of FAZ area and VD at DCP were 0.672 and 0.505, respectively, compared to 0.976 and 0.840 at SCP in previous reports [17, 42]. Nevertheless, the ICC values of our customized software were comparable with the built-in software using the same OCT-A device measuring the DCP metrics reported by a previous study [43]. The lower reliabilities in DCP may be explained by the fact that the FAZ at SCP and FAZ at DCP have different shapes and that its contours at DCP are less sharply defined [44, 45]. The current lower reliabilities in measuring FAZ area and VD may undermine OCT-A with regards to its use as a clinical tool for detecting DR changes in DCP. We acknowledge additional potential limitations of the current study, such as the limited view of 3 mm × 3 mm images (although 3 mm × 3 mm images have the advantage of increased resolution compared to larger scan sizes) [46], the use of a single subjective reader for OCT-A image quality control even with strict and objective criteria [47], caution in generalizing conclusions beyond the Chinese population used in the study, and the lack of mechanistic analysis inherent in cross-sectional clinical studies such as ours.

## Conclusion

The effects of ocular and systemic factors have to be considered in order to yield accurate and meaningful interpretations of diabetic changes in the retinal microvasculature identified in the images taken by OCT-A. Continuous efforts should be made to improve the quality of images and reliability of images produced by OCT-A to make it a useful tool to detect early retinal microvascular changes and to monitor and predict development and progression of DR among patients with DM.

## Supplementary information

**Additional file 1: Table S1.** Comparisons between eyes with/without projection artifacts

**Additional file 2: Figure S1.** Examples of images showing different morphologies between superficial capillary plexus (SCP, a-c) and deep capillary plexus (DCP, d-f) in diabetic eyes

## Data Availability

The datasets used and/or analyzed during the current study are available from the corresponding author on reasonable request.

## Abbreviations

AL: Axial length
BMI: Body mass index
CST: Central subfield thickness
DBP: Diastolic blood pressure
DCP: Deep capillary plexus
DM: Diabetes mellitus
DME: Diabetic macular edema
DMI: Diabetic macular ischemia
DR: Diabetic retinopathy
FAZ: Foveal avascular zone
FD: Fractal dimension
GC-IPL: Ganglion cell-inner plexiform layer
GEE: Generalized estimating eqs.
ICC: Intra-class correlation coefficients
ICP: Intermediate capillary plexus
INL: Inner nuclear layer
IPL: Inner plexiform layer
LDL: Low-density lipoprotein
LogMAR: Logarithm of the minimum angle of resolution
NPDR: Non-proliferative DR
PDR: Proliferative diabetic retinopathy
SBP: Systolic blood pressure
VD: Vessel density
