# Supplementary material for: Clinically relevant factors associated with quantitative optical coherence tomography angiography metrics in deep capillary plexus in patients with diabetes
Source: Eye Vis (Lond). 2020 Feb 3;7:7. doi: 10.1186/s40662-019-0173-y (PMC6996172; doi:10.1186/s40662-019-0173-y)
Supplement: Supplementary file 1 — Additional file 1: Table S1. Comparisons between eyes with/without projection artifacts [file 40662_2019_173_MOESM1_ESM.docx]

**Supplementary Table 1.** Comparisons between eyes with/without projection artifacts.

|  | **No projection artifacts**  **By eyes (n = 397)** | **Excluded due to projection artifacts**  **By eyes (n = 53)** | **P-value** |
| --- | --- | --- | --- |
| Diabetic retinopathy severity  (no/ mild/ moderate/ severe) | 170/ 101/ 190/ 36  (43%/ 25%/ 23%/ 9%) | 20/ 18/ 7/ 8  (38%/ 34%/ 13%/ 15%) | **0.022** |
| Presence of diabetic macular edema | 76 (14.07%) | 9 (14.06%) | 0.998 |
| LogMAR | 0.18 (0.18) | 0.26 (0.24) | **0.001** |
| Axial length (mm) | 23.91 (1.37) | 24.06 (1.45) | 0.423 |
| Thickness central subfield | 259.46 (50.59) | 259.81 (78.93) | 0.961 |
| Average ganglion cell inner plexiform layer thickness (µm) | 79.14 (11.34) | 77.55 (14.40) | 0.312 |
| ONH Ave RNFL (µm) | 95.53 (56.84) | 92.88 (17.90) | 0.710 |
| Subfoveal choroidal thickness (µm) | 202.35 (79.45) | 186.74 (66.11) | 0.141 |
|  | **By subjects (n = 250)** | **By subjects (n = 40)** |  |
|  |  |  |  |
| Gender, Female | 113 (45.20%) | 19 (47.50%) | 0.943 |
| Age (year) | 60.73 (13.20) | 65.67 (13.61) | **0.037** |
| Duration of diabetes (year) | 13.73 (10.48) | 12.98 (9.02) | 0.671 |
| Body mass index (kg/m^2^) | 25.28 (5.44) | 26.18 (4.06) | 0.320 |
| Systolic blood pressure (mmHg) | 137.60 (20.33) | 145.28 (21.53) | **0.040** |
| Diastolic blood pressure (mmHg) | 78.13 (10.71) | 74.05 (9.98) | **0.025** |
| Pulse Pressure (mmHg) | 60.44 (18.38) | 71.23 (17.82) | **0.001** |
| HbA_1C_ (%) | 7.49 (1.33) | 7.38 (1.52) | 0.691 |
| Fasting glucose (mmol/L) | 7.84 (3.35) | 7.80 (2.78) | 0.947 |
| Total cholesterol (mmol/L) | 4.06 (0.78) | 4.33 (0.95) | 0.101 |
| LDL cholesterol (mmol/L) | 2.29 (0.75) | 2.13 (0.70) | 0.223 |
| HDL cholesterol (mmol/L) | 1.37 (0.50) | 1.33 (0.43) | 0.645 |
| Creatinine (µmol/L) | 96.38 (94.21) | 107.08 (82.90) | 0.507 |
| History of Stroke | 9 (4.43%) | 5 (10.63%) | 0.127 |
| History of CAD | 35 (17.24%) | 13 (34.21%) | **0.016** |

DR= diabetic retinopathy; GC-IPL= ganglion cell inner plexiform layer; LogMAR= logarithm of the minimum angle of resolution; ONH= optic nerve head; Ave= average; RNFL= retinal nerve fiber layer; HbA_1C_= hemoglobin A1c; LDL= low-density lipoprotein; HDL= high-density lipoproteins; CAD= coronary artery disease
