# Supplementary figures and images for: Clinically relevant factors associated with quantitative optical coherence tomography angiography metrics in deep capillary plexus in patients with diabetes
Source: Eye Vis (Lond). 2020 Feb 3;7:7. doi: 10.1186/s40662-019-0173-y (PMC6996172; doi:10.1186/s40662-019-0173-y)

**Supplementary figure 1.**


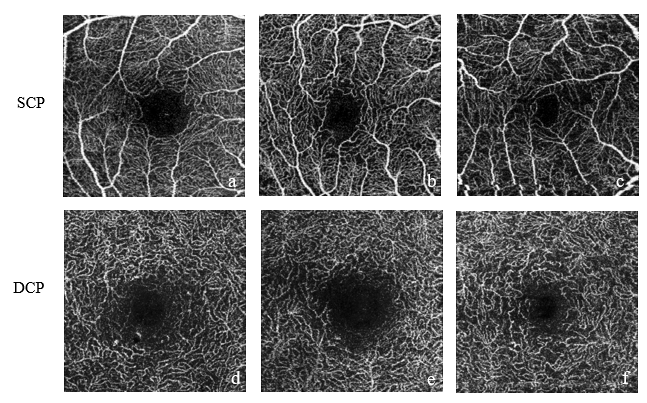

Supplement: Supplementary file 2 — Additional file 2: Figure S1. Examples of images showing different morphologies between superficial capillary plexus (SCP, a-c) and deep capillary plexus (DCP, d-f) in diabetic eyes [file 40662_2019_173_MOESM2_ESM.docx]
